# Age-Related Macular Degeneration Staging by Color Fundus Photography vs. Multimodal Imaging—Epidemiological Implications (*The Coimbra Eye Study—Report 6*)

**DOI:** 10.3390/jcm9051329

**Published:** 2020-05-02

**Authors:** Cláudia Farinha, Maria Luz Cachulo, Rita Coimbra, Dalila Alves, Sandrina Nunes, Isabel Pires, João Pedro Marques, José Costa, Amélia Martins, Isa Sobral, Patrícia Barreto, Inês Laíns, João Figueira, Luisa Ribeiro, José Cunha-Vaz, Rufino Silva

**Affiliations:** 1 AIBILI—Association for Innovation and Biomedical Research on Light and Image, 3000-548 Coimbra, Portugal; mluzcachulo@gmail.com (M.L.C.); racoimbra@aibili.pt (R.C.); dalves@aibili.pt (D.A.); sandrina@aibili.pt (S.N.); isabel.maravilha@sapo.pt (I.P.); marquesjoaopedro@gmail.com (J.P.M.); jfcosta@aibili.pt (J.C.); martins.amelia9@gmail.com (A.M.); isagabrielag@gmail.com (I.S.); pbarreto@aibili.pt (P.B.); joaofigueira@oftalmologia.co.pt (J.F.); lr@aibili.pt (L.R.); cunhavaz@aibili.pt (J.C.-V.); rufino.silva@oftalmologia.co.pt (R.S.); 2 Ophthalmology Department, Centro Hospitalar e Universitário de Coimbra (CHUC), 3004-561 Coimbra, Portugal; 3 Faculty of Medicine—University of Coimbra (FMUC), 3000-370 Coimbra, Portugal; 4 Massachusetts Eye and Ear, Harvard Medical School, Boston, MA 02114, USA; ineslains@gmail.com; 5 Coimbra Institute for Clinical and Biomedical Research, Faculty of Medicine, University of Coimbra (iCBR- FMUC), 3000-548 Coimbra, Portugal

**Keywords:** age-related macular degeneration, AMD staging, multimodal Imaging, early AMD, late AMD

## Abstract

Epidemiology of age-related macular degeneration (AMD) is based on staging systems relying on color fundus photography (CFP). We aim to compare AMD staging using CFP to multimodal imaging with optical coherence tomography (OCT), infra-red (IR), and fundus autofluorescence (FAF), in a large cohort from the Epidemiologic AMD Coimbra Eye Study. All imaging exams from the participants of this population-based study were classified by a central reading center. CFP images were graded according to the International Classification and Grading System for AMD and staged with Rotterdam classification. Afterward, CFP images were reviewed with OCT, IR, and FAF and stage update was performed if necessary. Early and late AMD prevalence was compared in a total of 1616 included subjects. In CFP-based grading, the prevalence was 14.11% for early AMD (*n* = 228) and 1.05% (*n* = 17) for late AMD, nine cases (0.56%) had neovascular AMD (nAMD) and eight (0.50%) geographic atrophy (GA). Using multimodal grading, the prevalence increased to 14.60% for early AMD (*n* = 236) and 1.61% (*n* = 26) for late AMD, with 14 cases (0.87%) of nAMD and 12 (0.74%) of GA. AMD staging was more accurate with the multimodal approach and this was especially relevant for late AMD. We propose that multimodal imaging should be adopted in the future to better estimate and compare epidemiological data in different populations.

## 1. Introduction

Age-related macular degeneration (AMD) is the leading cause of central vision loss in the elderly populations of industrialized countries [1] , and this burden is expected to increase as recent estimates point to a projected number of 288 million affected individuals by 2040 [2]. Epidemiologic studies on prevalence and incidence of AMD are therefore cornerstones for planning for demand in health care systems and for establishing preventing measures [3].

To date, several population-based studies have provided important information on AMD epidemiology [4,5,6,7,8,9,10]. However, classification and grading in AMD, which is the basis of epidemiologic studies, differs across them and there is still no consensus. Despite several attempts by study groups, no global classification system exists, and this is especially true for classification of early/ intermediate AMD [11,12,13,14,15]. Furthermore, as data obtained with different imaging methods provides further insight on AMD pathophysiology, further confounding factors in this matter arise. Novel and updated definitions at the level of AMD lesions are emerging and redefined [16,17]. In fact, we now witness a change in AMD grading from the conventional color fundus based classification approach to a multimodal one, capable of more accuracy in disease diagnosis and staging [17,18,19,20]. Implementation of this multimodal approach to epidemiologic studies will probably be the necessary next step, in order to precisely stage AMD and to achieve true comparability between populations. However, the extent of the discrepancies that would arise when implementing a multimodal classification, compared to the conventional color fundus photography (CFP) based grading, is not known in epidemiologic analysis. This is especially relevant if studies using novel multimodal-based staging systems are to be compared to older studies based only on CFP.

The Coimbra Eye Study (CES) was the first population-based study providing several reports on AMD prevalence and associated risk factors in Portugal (NCT01298674) [21,22,23,24]. The study included two distinct populations in central Portugal—Mira, a coastal town, and Lousã, an inland town. Recently, we conducted a follow-up study for the coastal town of Mira and we reported on AMD incidence over six years for this population (NCT02748824) [25]. All subjects who participated in this follow-up visit were submitted to multimodal imaging, alongside conventional color fundus photography (CFP).

The purpose of the present report is to compare the staging of AMD when using color fundus photography (CFP) grading only to grading using a multimodal approach with CFP, spectral-domain optical coherence tomography (SD-OCT), infra-red (IR), and fundus autofluorescence (FAF) imaging, and to analyze the consequent epidemiologic changes in the set of patients from the coastal town of Mira that participated in the AMD Incidence Coimbra Eye Study. 

## 2. Experimental Section

### 2.1. Study Design and Population

Information on the identification and description of the study population has been reported elsewhere [21,22,25]. Briefly, subjects who participated in the baseline analysis for the estimation of AMD prevalence in Portugal (NCT01298674) and recruited from the primary healthcare unit in Mira were identified and the surviving cohort was invited to participate in the 6.5-year follow-up analysis for the estimation of AMD incidence (NCT02748824) [25].

Signed informed consent was obtained for all participants, the study adhered to the tenets of the Declaration of Helsinki (2008) and was approved by the Association for Innovation and Biomedical Research on Light and Image (AIBILI) Ethics Committee. 

### 2.2. Data Collection and Ophthalmic Examination

All participants underwent a detailed questionnaire-based interview on demographic, clinical, and lifestyle related information, as well as complete bilateral ophthalmological examination including best-corrected visual acuity (BCVA), tested with Early Treatment Diabetic Retinopathy Study (ETDRS) charts.

Color fundus photographs were obtained after pharmacological mydriasis. Fields 1M (centered on the optic disc), 2 (centered on the macula), and 3M (temporal to the macula) acquired at 45° for both eyes, were recorded using a digital Topcon® fundus camera (TRC-NW8; Topcon Corp., Tokyo, Japan). Fundus reflex photographs were taken to document media opacities. 

Spectral-domain optical coherence tomography, fundus autofluorescence, and infrared imaging of both eyes were also acquired with Spectralis HRA+OCT (Heidelberg Engineering, Heidelberg, Germany). SD-OCT acquisitions consisted of one EDI Macular Volume Scan (20° × 20°, 49 B-scans, 16 frames per scan), 1 radial scan centered in the fovea (20° × 20°, 24 B-scans, 10 frames per scan) and 2 high resolution EDI Line Scans (30°, acquired at 0° and 90°, with ≥20 frames each), with signal strength ≥25. Both FAF (488 nm) and IR images were acquired for field 2 at 30° (High Resolution with ≥15 frames each).

### 2.3. Grading Methods 

All imaging exams were sent to a centralized reading center for grading (Coimbra Ophthalmology Reading Center—CORC, AIBILI). CFP images were exported in TIFF format and OCT, FAF, and IR exams were exported as Heidelberg E2E files. All graders were ophthalmologists certified by the reading center according to the specific study protocol. All exams were analyzed and graded in a stepwise manner. Images were defined as gradable if they complied with established quality criteria by the reading center (e.g., sufficient brightness and color contrast, full macular region visible, OCT scans with good signal and well centered).

First, a general analysis of all included participants was carried out to identify major retinal pathology and to identify suspected AMD cases. Images were excluded from AMD differential grading if they had obscuring lesions (e.g., cataract) or lesions from other concomitant retinal disease hampering correct AMD grading. This analysis was independently performed by the graders, however, a second senior grader performed additional independent grading of 2% of cases. Discrepancies were analyzed and final reports were sent for the participant and his primary care physician at the healthcare unit. 

After this preliminary grading, 4 senior medical retina specialists graders (C.F., M.L.C., I.P., J.P.M.) performed a differential analysis and graded the suspected AMD cases using the International Classification and Grading System (ICGS), and subsequently they staged for severity using the Rotterdam classification [12,26].

The differential grading was performed in a two-step approach as follows. First, grading was performed by grading CFP images alone. This was done to allow for comparability with our prevalence study that was based in CFP only [21,22]. CFP image quality was adjusted using color balance software to automatically standardize the brightness, contrast, and color [27]. Image grading was supported by Retmarker AMD Research software (Retmarker SA, Coimbra, Portugal), that assists manual grading of lesions according to the International Age-Related Macular Epidemiological Study Group Classification [12,28]. Afterwards, the graders classified the signs of AMD into 5 exclusive stages (stage 0–4) using the Rotterdam staging system (Table 1) [7,26]. Staging of an individual participant was based on the eye with more severe status if both eyes were gradable, and on the gradable eye if only one eye was gradable. 

After this first step, the graders opened for each case the correspondent SD-OCT, IR, and FAF images in the Heidelberg Eye Explorer software, and all CFP images were reviewed with grading now being performed in a multimodal manner. A new updated stage was recorded whenever necessary. 

A second senior grader performed an independent grading of all late AMD cases and of 5% the remaining cases. Consensus was achieved after discussion between the two graders, and if that could not be attained a third senior adjudicator grader oversaw the final decision. 

### 2.4. Disease Definitions and AMD Classification

Early AMD was defined by the presence of large (≥125 µm in diameter), soft, indistinct, or reticular drusen only; or of soft distinct (≥63 µm in diameter), indistinct (≥125 µm), or reticular drusen with pigmentary abnormalities, within the macula, in the absence of signs of late AMD. This definition corresponds to stage 2 and 3 in the Rotterdam classification [7,26]. Participants with only distinct soft drusen or retinal pigmentary abnormalities (stage 1), were not defined as early AMD.

Late AMD was defined by the presence of neovascular AMD (nAMD) or geographic atrophy (GA) within the grid (3000 µm from the center of the fovea). In CFP analysis, nAMD included serous or hemorrhagic detachment of the retinal pigment epithelium (RPE) or sensory retina, intra, subretinal, or sub-RPE hemorrhages and fibrous scar. GA was defined as an area of retinal depigmentation (≥175 μm), characterized by sharp borders and visualization of choroidal vessels [4,5,12]. In multimodal analysis, nAMD was considered present if there were signs of any type 1, 2, and 3 neovascularization with associated features of intra/sub-retinal fluid, hemorrhage, and/or sub-retinal fibrosis. For GA to be considered present in multimodal analysis an appearance of complete RPE and outer retina atrophy (cRORA) must have been present in OCT, and a corresponding sharply demarcated area of deep hypoautofluorescence in FAF imaging was required [16,17]. When GA and nAMD coexisted in the same eye, it was categorized as nAMD. Late AMD corresponds to stage 4 in the Rotterdam classification [7].

### 2.5. Statistical Analysis 

Descriptive statistics were used to describe the prevalence of AMD in the follow-up population in terms of the Rotterdam classification, and to explore the differences in AMD stage between the two methodologies (CFP vs. multimodal imaging). Both crude and age-standardized prevalence of early and late AMD were calculated. Age-standardization was performed for the Portuguese population according to Census 2011 of the National Institutes of Statistics (www.ine.pt). Cumulative incidence was calculated for each grading method in order to further explore for differences. Data were analyzed using STATA software (StatCorp., College Station, TX, USA), version 12.1 SE. 

## 3. Results

In the scope of the Coimbra Eye Study, 1617 subjects from the coastal town of Mira were included in the AMD incidence study [25]. Briefly, in the 6.5-year follow-up examination there were 2975 eligible participants, of whom 536 had died or were bedridden and 822 who did not participate. After excluding one participant because of ungradable imaging exams of both eyes, the final cohort of 1616 participants was achieved. The mean age of this population was 72.5 years old (sd. 6.83 range. 60.3–91.9). 

After the general grading of the 1616 included participants, 460 were classified as suspected of having any stage of AMD and differential grading with CFP followed by multimodal grading was carried out, as stated above. 

For the comparative analysis between grading methods (CFP vs. multimodal), 457 participants were included, as three participants were excluded—two participants because CFP did not have enough quality for lesion grading, and one because OCT, FAF, and IR imaging was of low quality for accurate grading. 

### 3.1. Stage Frequency by Grading Method

**Figure 1:**
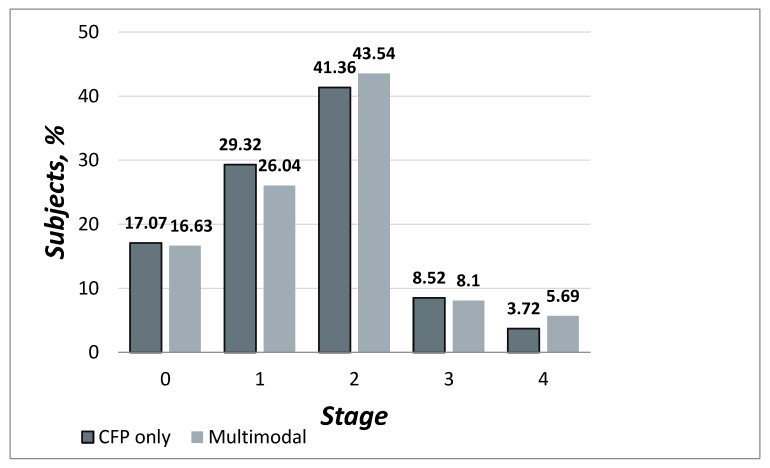
Frequency distribution of differential grading cases, by stage and grading approach.

The stage frequency distribution by severity and by grading modality is presented in Table 2 and Figure 1. Classification as stage 2a (soft indistinct drusen ≥ 125µm or reticular drusen) and as stage 4 (GA and/or nAMD) was more frequent in multimodal grading compared to CFP based grading, while all other stages were less prevalent using multimodal approach.

The differences and changes in the stages of all participants included in differential analysis are shown in detail in Table 3. The increase in stage 2a prevalence in multimodal grading is due to misclassification in CFP as stage 1a (*n* = 14) and stages 0a and 0b (*n* = 4). The increase in stage 4 in multimodal grading compared to CFP (*n* = 26 vs. *n* = 17) is due to misclassification in CFP as stages 3 (*n* = 5), 2 (*n* = 3) and 1b (*n* = 1). Participants classified as stage 3 in CFP (*n* = 39) maintained that stage in multimodal in 33 cases, but five were reclassified as stage 4 in multimodal evaluation and one to stage 0a (no AMD). 

Globally, CFP mostly underestimated the presence of disease, that is, early and late AMD. There were only seven cases (1.53%) misclassified as the superior stage in CFP and regraded to lower stages in the multimodal approach. In addition, only two of these (0.44%) were misclassified as stage 2a— that is, early AMD—in CFP and reclassified to 1a, or no disease, in multimodal. 

### 3.2. Causes of stage update—CFP vs. Multimodal

The increase in stage 2a prevalence with multimodal imaging was mainly due to the more accurate detection of reticular drusen or pseudodrusen. Of the 18 cases misclassified as lower stages in CFP, we observed that in 14 of these pseudodrusen not clearly seen in CFP were detected in OCT, FAF and IR imaging, and consequently the stage was updated to 2a, this is from “no disease” to “early AMD”. 

Other causes identified for change in stage were drusen initially graded as present on CFP but absent on multimodal, or vice-versa. This was mainly due to: image quality interfering with accurate grading; presence of other drusen-like lesions such as vitelliform deposits or changes in the vitreoretinal interface that mimicked drusen appearance in CFP; presence of drusen-like lesions in CFP without any change visible in OCT/FAF/IR; and presence of single drusen on OCT not seen in CFP.

Concerning late AMD, we found that stage 4 was misclassified as a lower stage in nine participants when using only CFP, and this was found to be related with early cases of nAMD or GA not clearly seen in fundus images, or due to CFP image quality preventing discrimination of fine details.

### 3.3. Prevalence of Early and Late AMD

Considering the 1616 included participants of the Mira population in the follow-up visit, we found that using CFP grading only, the crude prevalence was 14.11% for early AMD (*n* = 228) and 1.05% (*n* = 17) for late AMD, with nine cases (0.56%) of neovascular AMD and eight (0.50%) of geographic atrophy. When using the multimodal approach, the crude prevalence increased to 14.60% for early AMD (*n* = 236) and 1.61% (*n* = 26) for late AMD. There were 14 detected cases (0.87%) of nAMD and 12 cases (0.74%) of GA. Crude and age-standardized prevalence to the Portuguese population is presented in Table 4.

Globally, the difference between the prevalence of AMD (stages 2, 3, and 4) based only in CFP and based in the multimodal approach was 1.42% (95% CI, 0.90%–2.14%). When analyzing the cases with early AMD only the difference was 1.9% (95% CI, 1.3%–2.7%); and for late AMD only the difference between the two methods was 0.5% (95% CI, 0.3%–1.1%).

### 3.4. Incidence of Early and Late AMD

Considering those cases who performed both CFP grading and multimodal grading and our previous report on AMD prevalence in the CES by Cachulo ML et al. [22], the 6.5-year cumulative incidence was found to be 10.69% for early AMD (*n* = 158) and 0.75% (*n* = 12) for late AMD when using CFP grading only. However, if the multimodal approach is used at follow-up, the cumulative incidence changes to 11.03% for early AMD (*n* = 163) and 1.31% (*n* = 21) for late AMD.

## 4. Discussion

The AMD incidence study from the Coimbra Eye Study was the first population-based study to investigate the long-term incidence of AMD in Portugal [25]. It is also one of the few that explores and compares AMD staging when accessed in a multimodal manner to that obtained with CFP only, which is to date the basis of major epidemiological studies [6,29]. Our results show that there are important differences in staging when using multimodal grading, with an increase in prevalence of both early and late AMD. These differences were mainly due to more accuracy of multimodal imaging in the detection of pseudodrusen in early AMD and incipient cases of nAMD or GA. We also found that cumulative incidence is higher, and probably overestimated, if follow-up visits in longitudinal studies are switched to multimodal grading when CFP only was used at baseline.

In our study, in which the Rotterdam classification was used, CFP grading generally underestimated the stage of AMD relative to that determined with multimodal imaging. This was observed in early AMD, with change from lower “no disease” stages to stage 2a, and in late AMD, with cases being updated mainly from stage 3 to stage 4. This is an important finding because it translated to an increase in prevalence of early AMD and of late AMD in our cohort when using a multimodal approach. 

We cannot extrapolate as to how early AMD stage and prevalence would be affected in other classification systems such as the more recent Beckman classification, as for instance reticular pseudodrusen are not considered in this classification for staging purposes [14,29]. Even so, a recent preliminary report from the NICOLA study using the Beckman system revealed multimodal grading to be superior in the detection of AMD lesions compared to CFP alone, and to significantly influence staging [29]. Other classification systems do consider reticular pseudodrusen, but introduce other important differences, such as drusen area and other definitions, that makes comparisons impossible [15,30]. Even the definition of “early AMD” per se differs across classifications [14,15,26,31]. However, in cohorts classified with the Rotterdam system, our findings provide an important notion that early AMD might be underrepresented when using CFP only compared to multimodal imaging, and groups using this classification should be aware of these differences and more analysis on the subject should be conducted [8,9,10].

As for late AMD, the global definition is more consensual and therefore our results show an underestimation of prevalence when using conventional CFP-based classification are more readily extrapolated to other studies. Crude prevalence of late AMD using CFP only was 1.05% in our cohort, however, it increased to 1.61% with multimodal imaging (1.81% if age-standardized to the Portuguese population). 

This change affected both late AMD forms—nAMD and GA—with maintenance of the nAMD/GA proportion. This means that there is a possibility that in major prevalence studies late AMD is also underestimated, and both GA and nAMD are more prevalent than commonly thought. The ALIENOR study [6] also reported on differences in late AMD when OCT was introduced in the grading. They reported on 34 participants with incident late AMD with grading based only in CFP, but when SD-OCT was added, the number increased to 45 participants. In this regard, Colijn J et al. [9] also acknowledge in their metanalysis of AMD prevalence in Europe that an important limitation of the estimates presented is that classification of AMD used in the included studies was based in CFP only, and as multimodal imaging better visualizes edema and subtle changes resulting from choroidal neovascularization (CNV), their data might have underestimated the true prevalence of CNV. As for GA, it is now increasingly recognized that both FAF and OCT are recommended for diagnosis and detection of atrophy progression, and therefore the higher detection rate of GA when using multimodal imaging in our study was not unexpected [16,17].

The accuracy of CFP in late AMD diagnosis seems therefore below optimal, and we demonstrated an important difference of stage 4 diagnosis between methods. Multimodal imaging shows superior accuracy in this particular setting and we believe that this approach should be considered the gold standard in the future in epidemiologic studies. Greater diagnostic accuracy might even mitigate significant discrepancies on prevalence and incidence between populations from whom CFPs were graded and analyzed in different conditions.

Based on the more complete information provided by multimodal imaging, Spaide [19] recently proposed a multimodal classification system for AMD that aims to incorporate the diverse spectrum of AMD presentations in order to better predict outcomes. This approach considers soft drusen, but also pachydrusen and pseudodrusen as well as choroidal features and associates them with progression towards specific phenotypes of late AMD. For example, thinner choroids are more likely to present with pseudodrusen and type 3 neovascularization, while AMD eyes with thicker choroids are at greater risk of type 1 CNV, including polypoidal choroidal vasculopathy. Therefore, the understanding that AMD is better characterized by multimodal imaging is essential, and it must be extended to classification systems and epidemiology studies. 

Another finding in our study was that with multimodal grading the incidence rate of early and late AMD was also different. For early AMD, the 6.5-year cumulative incidence was 11.03% (*n* = 163) with multimodal grading and 10.69% (*n* = 158) with CFP. As for late AMD, the incidence was 1.31% (*n* = 21) with multimodal and 0.75% (*n* = 12) with CFP. Although there is a chance that the incidence is truly higher when using multimodal imaging, this probably represents an overestimation for using a more disease and stage-sensitive method in the follow-up analysis. Disease burden at baseline would also be expected to change in the same proportion if OCT was used at this time-point. Therefore, if multimodal imaging is to be used in older stablished cohorts analyzed only with CFP at baseline, this overestimation bias in incidence must be kept in mind. 

This study has several limitations. First, our follow-up visit cohort was reduced to 54.4% of the original one and this might have introduced a selection bias due to selective follow-up and mortality. However, the final population in analysis for differences in grading methodology on prevalence is still large, and important differences were readily seen. Another bias could be that we did not a corrected prevalence for participants with only one gradable eye. These could be misclassified towards a lower stage if the ungraded eye is worse than the observed one. However, the main purpose of the study was to compare between different grading methods and to analyze their impact in staging. 

The strengths of our study are the relatively long-term follow-up of a population-based cohort, the use of a validated AMD grading system used in major epidemiologic studies, and we are one of the first studies extensively addressing prevalence and incidence of early and late AMD with different grading methodologies—CFP vs. multimodal. The utilization of multimodal grading allowed us to better detect AMD lesions such as pseudodrusen, incipient neovascular lesions, or small areas of GA. Therefore, we provide a more accurate report on early and late AMD prevalence in a large cohort from a Portuguese population, as well as of the impact that introduction of multimodal imaging carries in the epidemiologic context of AMD.

In summary, we present the first report in a populational study truly comparing AMD staging with Rotterdam classification using CFP versus multimodal imaging grading. AMD staging proved to be more accurate using the multimodal approach in our study and this was especially true for the correct identification of late AMD, where the difference between the two methods is critical. We believe that multimodal imaging should be adopted in future AMD classification systems and epidemiologic studies, in order to better estimate and compare AMD prevalence and incidence in different populations.

## Figures and Tables

**Table 1:** Classification of mutually exclusive stages of age-related macular degeneration (AMD) according to the Rotterdam staging system.

Stage	Definition
**0**	
**a**	No signs of AMD at all
**b**	Hard drusen (<63 µm) only
**1**	
**a**	Soft distinct drusen (≥63 µm) only
**b**	Pigmentary abnormalities only, no soft drusen (≥63 µm)
**2**	
**a**	Soft indistinct drusen (≥125 µm) or reticular drusen only
**b**	Soft distinct drusen (≥63 µm) with pigmentary abnormalities
**3**	Soft indistinct (≥125 µm) or reticular drusen with pigmentary abnormalities
**4**	Atrophic or neovascular AMD

**Table 2:** Frequency distribution of the mutually exclusive stages of AMD by grading methodology, according to the Rotterdam classification.

*n* = 457	CFP only	%	Multimodal	%
**0a**	20	4.38	19	4.16
**0b**	58	12.69	57	12.47
**1a**	126	27.57	113	24.73
**1b**	8	1.75	6	1.31
**2a**	171	37.42	184	40.26
**2b**	18	3.94	15	3.28
**3**	39	8.53	37	8.10
**4**	17	3.72	26	5.69

Stage based in worst eye, if both were gradable; CFP—color fundus photography

**Table 3:** Crosstab of the distribution of AMD stages based in CFP only and based in the multimodal approach.

**CFP only**	**Multimodal**									
		**0a**	**0b**	**1a**	**1b**	**2a**	**2b**	**3**	**4**	**Total**
	**0a**	17	0	0	1	2	0	0	0	20
	**0b**	0	54	1	1	2	0	0	0	58
	**1a**	1	2	109	0	14	0	0	0	126
	**1b**	0	1	1	4	0	0	1	1	8
	**2a**	0	0	2	0	166	0	2	1	171
	**2b**	0	0	0	0	0	15	1	2	18
	**3**	1	0	0	0	0	0	33	5	39
	**4**	0	0	0	0	0	0	0	17	17
	**Total**	19	57	113	6	184	15	37	26	457

**Table 4:** Comparison of early and late AMD prevalence based in CFP versus multimodal approach.

Prevalence %	Early AMD	Late AMD	nAMD	GA	Early AMD Age-Standardized	Late AMD Age-Standardized
**CFP**	14.11 (*n* = 228)	1.05 (*n* = 17)	0.56 (*n* = 9)	0.50 (*n* = 8)	13.36% (95% CI, 11.64%–15.27%)	1.23% (95% CI, 0.76%–1.91%)
**Multimodal**	14.60 (*n* = 236)	1.61 (*n* = 26)	0.87 (*n* = 14)	0.74 (*n* = 12)	13.84% (95% CI, 12.11%–15.80%)	1.81% (95% CI, 1.20%–2.58%)

nAMD—neovascular AMD; GA—geographic atrophy.
